# Global prevalence of self-harm during the COVID-19 pandemic: a systematic review and meta-analysis

**DOI:** 10.1186/s40359-023-01181-8

**Published:** 2023-05-05

**Authors:** Huan Cheng, Di Wang, Lu Wang, Haiou Zou, Yanhua Qu

**Affiliations:** 1 Department of Nursing, Peking University First Hospital, No. 8 Xi Shi Ku Street, Xi Cheng District, Beijing, 100034 China; 2 School of Nursing, Anhui Medical University, No. 69 Mei Shan Road, ShuShan District, Hefei, 230031 Anhui Province China; 3 School of Nursing, Chinese Academy of Medical Sciences & Peking Union Medical College, No. 33 Badachu Road, Shi Jing Shan District, Beijing, 100144 China; 4 Department of Nursing, Beijing HuiLongGuan Hospital, Nandian Road, Chang Ping District, Beijing, 102208 China

**Keywords:** MERS-CoV, SARS-CoV-2, 2019-nCoV, Self-harm, Prevalence, Systematic review

## Abstract

**Background:**

COVID-19 and its transmission mitigation measures have caused widespread mental health problems. Previous studies have suggested that psychological, economic, behavioral, and psychosocial problems associated with the COVID-19 pandemic may lead to a rise in self-harm. However, little is known about the prevalence of self-harm worldwide during COVID-19. Therefore, a quantitative synthesis is needed to reach an overall conclusion regarding the prevalence of self-harm during the pandemic.

**Methods:**

By using permutations of COVID-19, self-harm or relevant search terms, we searched the following electronic databases from November 2019 to January 2022: Web of Science, PubMed, MEDLINE, Embase, PsycINFO, Cochrane Database of Systematic Reviews, China National Knowledge Infrastructure (CNKI), Wanfang Database and systematically reviewed the evidence according to MOOSE guidelines. We employed Cochran’s chi-squared test (Cochran’s Q), *I*^2^test and subgroup analysis to assess and address the heterogeneity. Sensitivity analysis was conducted by eliminating each included study individually and then combining the effects.

**Results:**

Sixteen studies that met the inclusion and exclusion criteria were identified, with sample sizes ranging from 228 to 49,227. The methodological quality of the included studies was mostly at the medium level. By using a random effect model, the pooled prevalence of self-harm was 15.8% (95% CI 13.3–18.3). Based on subgroup analysis, the following characteristics of the included studies were more likely to have a higher prevalence of self-harm: studies conducted in Asia or prior to July 2020, cross-sectional studies, samples recruited in hospitals or schools, adolescents, females, the purpose of self-harm (NSSI), mental symptoms and restriction experiences.

**Conclusions:**

We provided the first meta-analytic estimated prevalence of self-harm based on a large sample from different countries and populations. The prevalence of self-harm during COVID-19 was not encouraging and requires attention and intervention. Further high-quality and prospective research are needed in order to determine the prevalence of self-harm with greater accuracy because to the clear heterogeneity across the included studies. In addition, this study also provides new directions for future research, including the identification of high-risk groups for self-harm, the formulation and implementation of prevention and intervention programs, and the long-term impact of COVID-19 on self-harm.

**Supplementary Information:**

The online version contains supplementary material available at 10.1186/s40359-023-01181-8.

## Introduction

The severe acute respiratory syndrome coronavirus 2 (SARS-CoV-2) pandemic was found in China in late 2019 and spread rapidly worldwide [1]. On January 30, 2020, the International Health Regulations (2005) Emergency Committee declared the COVID-19 outbreak a Public Health Emergency of International Concern [2]. Since its emergence in December 2019, COVID-19 has caused an estimated 286 million confirmed cases and 5.4 million deaths worldwide at the time of writing [3]. The pandemic of COVID-19 poses a threat not only to the physical health of individuals, but also a direct or indirect burden to mental health.

The mental health of hundreds of millions of people has been affected by the response of individuals and the government to this major public health emergency, which has changed people's social, work, study and lifestyle [4]. On the one hand, the limited awareness of COVID-19 and the prevalent trend of COVID-19 have reduced people's belief in their own health and increased their concerns about maintaining health [5], worrying about being infected with COVID-19, and aggravating psychological stress [6]. On the other hand, the ongoing spread of COVID-19 among the majority of the global population has produced a situation in which many factors affecting mental health are also impacted, including physical/social distancing restrictions, full lockdown of cities, closure of schools and enterprises, loss of livelihood, reduction of economic activities, and shifting priorities of governments in their attempt to control COVID-19 outbreaks [7, 8].

As mentioned above, the measures taken by individuals and governments to ddress the epidemic may provide favorable conditions for the emergence of mental ealth problems. Emerging studies have investigated the effects of COVID-19 on a range of mental health problems, including anxiety, sleep disorders, depression, stress-related disorders, and even self-harm and suicide [9, 10]. As the major public health concerns relate to COVID-19, self-harm and suicide have been voiced concerns by many experts. Sahoo et al. [11] found that the rate of self-harm and suicide may increase since the epidemic due to the lack of social interaction and the increase in community anxiety. Similarly, other researchers believed that the psychological, economic, behavioral and social problems caused by COVID-19 may lead to a higher prevalence of self-harm and suicide [12]. However, Isumi’s [13] study suggested that the first wave of the COVID-19 pandemic has not significantly affected the rates of self-harm and suicide among children and adolescents. Therefore, this not only indicates that there are significant inconsistencies among the empirical studies but also suggests that the fluctuation characteristics of COVID-19, study population or regional differences may affect the prevalence of self-harm and suicide.

Suicide, which was connected to the following factors, was not included in our research since we were only interested in self-harm. First, the understanding of self-harm/suicide during COVID-19 is limited at present, especially self-harm. Compared with suicide, there were few studies that analyzed self-harm separately, but mixed self-harm with suicide [14], making it difficult to obtain separate information on self-harm. Self-harm is not equal to suicide. The former can be divided into self-harm with and without suicide intention [15]. Considering the physical and psychological damage of self-harm to individuals, it is necessary to conduct a separate analysis of self-harm. Second, few studies have investigated the prevalence of self-harm during COVID-19, let alone systematic reviews. To date, all relevant studies have not been combined to quantitatively clarify the prevalence of self-harm during COVID-19, and little is known about the estimated prevalence of post-COVID-19 self-harm. Third, considering that the negative sequelae of COVID-19 (e.g., self-harm) are not evenly or randomly distributed in the population or place [16], the prevalence of self-harm in different regions and populations may be different. Comprehensive and comparable data would be available through the combined subgroup analysis of the study. Not only population and place differences but also the impact of differences in study time, study design or other characteristics on the prevalence of self-harm during COVID-19 can be obtained. Finally, the resources available to mitigate the impact of a pandemic on mental health and well-being are far from adequate at this stage [17], so the initiative and enthusiasm of countries and governments to deal with self-harm during the epidemic are limited.

Therefore, estimating the prevalence of self-harm is particularly important for strengthening attention to self-harm, maintaining physical and mental health, comprehensively assessing the burden, allocating resources reasonably and formulating targeted policies. The purpose of this study was to estimate the global prevalence of self-harm during COVID-19 in the form of a meta-analysis to identify its prevalence early and take measures to cushion the negative impact of the epidemic on self-harm.

## Methods

The design of this study was in line with the Meta-analysis of Observational Studies in Epidemiology (MOOSE) guidelines [18]. Based on this, research evidence of self-harm related to COVID-19 was systematically evaluated to determine the prevalence of self-harm during the COVID-19 pandemic. A protocol defining the key methodological parameters was developed and was registered at the International Platform of Registered Systematic Review and Meta-analysis Protocols (INPLASY), with registration number INPLASY202320049.

### Search strategy and screening

Two reviewers (LW & HC) with evidence-based experience were retrained in literature search and evidence-based medicine and were fully equipped to be proficient in the use of medical databases before starting a formal literature search. Two reviewers (LW & HC) independently searched for studies published from November 2019 to January 2022 that reported on the prevalence of self-harm related to COVID-19 without geographical restrictions in eight databases, including English databases (Web of Science; PubMed; MEDLINE; Embase; PsycINFO; Cochrane Database of Systematic Reviews) and Chinese databases (China National Knowledge Infrastructure-CNKI, Wanfang Database). A search strategy based on the following key search terms was used to identify the relevant literature: “Coronavirus” OR “Sars-Cov-2" (all variants), “self-harm” OR “self-injurious behavioral” OR “self-mutilation” (all variants). Truncations and related terms were used as appropriate based on individual database procedures (please see Additional file 1 for full search strategy). The search was last updated in January 2022. Through a manual search, the reviewers searched the list of references of the reviews related to the theme and other nonpublic Chinese conference literature to avoid omitting any meaningful studies.

We included all studies that reported individuals in any setting who reported self-harm during the COVID-19 pandemic. There were no age restrictions applied. We included individuals of any age who harm themselves with COVID-19-related adversity, whether for suicidal or nonsuicidal purposes. We included studies that described the measurement of self-harm using a validated measure and provided adequate information that could calculate the prevalence of self-harm in a population of interest. We included any means of self-harm that occurred during the COVID-19 pandemic.

We included all studies of individuals who reported self-harm during the COVID-19 pandemic. We excluded any studies that provided separate self-harm information that could not be obtained because it was mixed with suicide. We excluded any studies that reported only scores or extent of self-harm and thus could not obtain the prevalence. We excluded reviews, case reports or expert opinions and duplicate or overlapping data.

After completing the search process, all records were imported into EndNote X7 for independent screening by two reviewers (LW & HC) on titles and abstracts. Then, the examination of full text articles for inclusion was completed by two reviewers (LW & HC) independently. If full text was not available or there was insufficient information, one of the two reviewers would correspond with researchers as highlighted in the MOOSE guidelines, including contacting other researchers who jointly publish papers, contacting familiar researchers who work in the same institution, etc. Disagreements regarding study selection were resolved by consensus or, where appropriate, by a third reviewer (YQ).

### Data extraction and collection

Data extraction was completed independently by two reviewers (LW & HC) trained in data extraction. The key to data extraction was to identify the information about the prevalence of self-harm mentioned in the studies from the full text. Generally, the prevalence of self-harm is usually found in the method or result sections, which can be obtained directly or calculated according to the amount of self-harm given. If necessary, one of the two reviewers would correspond with the authors of the included study for more details.

Based on the prespecified outcomes, a data extraction sheet (designed by Excel 2010) was piloted and iteratively amended to improve relevant data capture. Relevant data were independently extracted by two reviewers (LW & HC) and cross-checked by another reviewer (YQ). Relevant data were extracted, including the first author’s name; year of publication; country; study time; study design; sample source; assessment tool; age of participants; sample size (male/female); and number and estimated prevalence of self-harm. For stratification variables, gender, mental symptoms and restrictions in individual studies were also extracted and coded. For the cohort studies, we extracted data on self-harm that occurred during COVID-19. For different studies with significant suspected overlapping data, the study with the largest sample size or the most comprehensive self-harm information prevailed. Epidata 3.1 software was used to perform the data entry.

### Quality assessment

Prior to the quality assessments, we ensured the greatest consistency among reviewers by ensuring that all reviewers involved in the assessment had experience in quality assessment and received unified training. The included papers were resent to the reviewers in printed and electronic form, with each paper hiding the information that affected the reviewers' objective judgment, including the journal name, author's name and work unit. The quality of the studies was assessed independently by two reviewers (LW & YZ) and reached consensus, with with a third reviewer resolving discrepancies through discussion and adjudication (DW).

Methodological quality assessment of the observational study used 11-item checklists recommended by the Agency for Healthcare Research and Quality (AHRQ), which includes data source, sample inclusion, bias, missing data, follow-up, etc. A [19]. The answers to 11 items were “yes”, “unclear” or “no”, answering “yes” to score 1 point and 0 points for the rest. Scores of 0–3, 4–7 and 8–11 were rated as high-, medium-, and low-quality studies, respectively [20]. For cohort studies, the Newcastle‒Ottawa Scale (NOS) was used to assess the methodological quality, which includes the selection of the study population, comparability, exposure or outcome evaluation. The total score of the NOS was between 0 and 9 points, and studies with scores below 5 points were considered to be of low quality [21].

### Statistical analysis

We conducted a meta-analysis of the prevalence of self-harm. These studies provided data on the percentage of participants who reported self-harm. The pooled prevalence with a 95% confidence interval (CI) was reported. Generally, in the meta-analysis of prevalence, if the prevalence of many studies reported is between 1 ~ 0.3 or 0.7 ~ 1, the weights of individual studies should be considered, and the transformed double arcsine method should be utilized [22]. Cochran’s chi-squared test (Cochran’s Q) and *I*^2^ test were used to analyze heterogeneity among the studies, with *P* < 0.1 or *I*^2^ > 75% signifying considerable heterogeneity. Given the diversity of the included studies, we expected some degree of heterogeneity between studies. Based on a literature review and clinical experience, we extracted many characteristics that may affect heterogeneity in the included studies, including study place, study time, study design, sample source, age of participants and purpose of self-harm. Individually, self-harm can be divided into self-harm with the purpose of dying and without the purpose of dying (non-suicidal self-injury, NSSI) [15], and the latter was separately grouped because they were separately identified in some included studies. Considering the stress that may be caused by the uncertainty, severity, and persistence of COVID-19 in the early stage, we classified the study time (Prior to/After July 2020). The study location was determined according to the continent of the country of the included studies. The above possible variables were analyzed by calculating the estimated values of each subgroup and the corresponding 95% CI. In addition, subgroup analysis was performed based on the stratified variables included in the study, including sex, mental symptoms and restrictions. Restrictions refer to restrictive measures related to COVID-19, including lockdown and physical distance measures. In addition, the robustness and reliability of the combined results of the meta-analysis were evaluated by eliminating each included study one by one and then combining the effects for sensitivity analysis. All analyses were performed by STATA 15.0. *P* values < 0.05 were identified as statistically significant for all tests.

## Results

### Study identification and selection

Initially, 731 records were returned through database search and manual search. A total of 729 records were returned by database search, including 122 records from MEDLINE, 115 records from Embase, 52 records from PsycINFO, 85 records from Web of Science, 123 records from PubMed, 18 records from Cochrane Database of Systematic Reviews, 112 records from CNKI and 79 records from Wanfang Database. We obtained 2 records through a manual search.

**Fig. 1:**
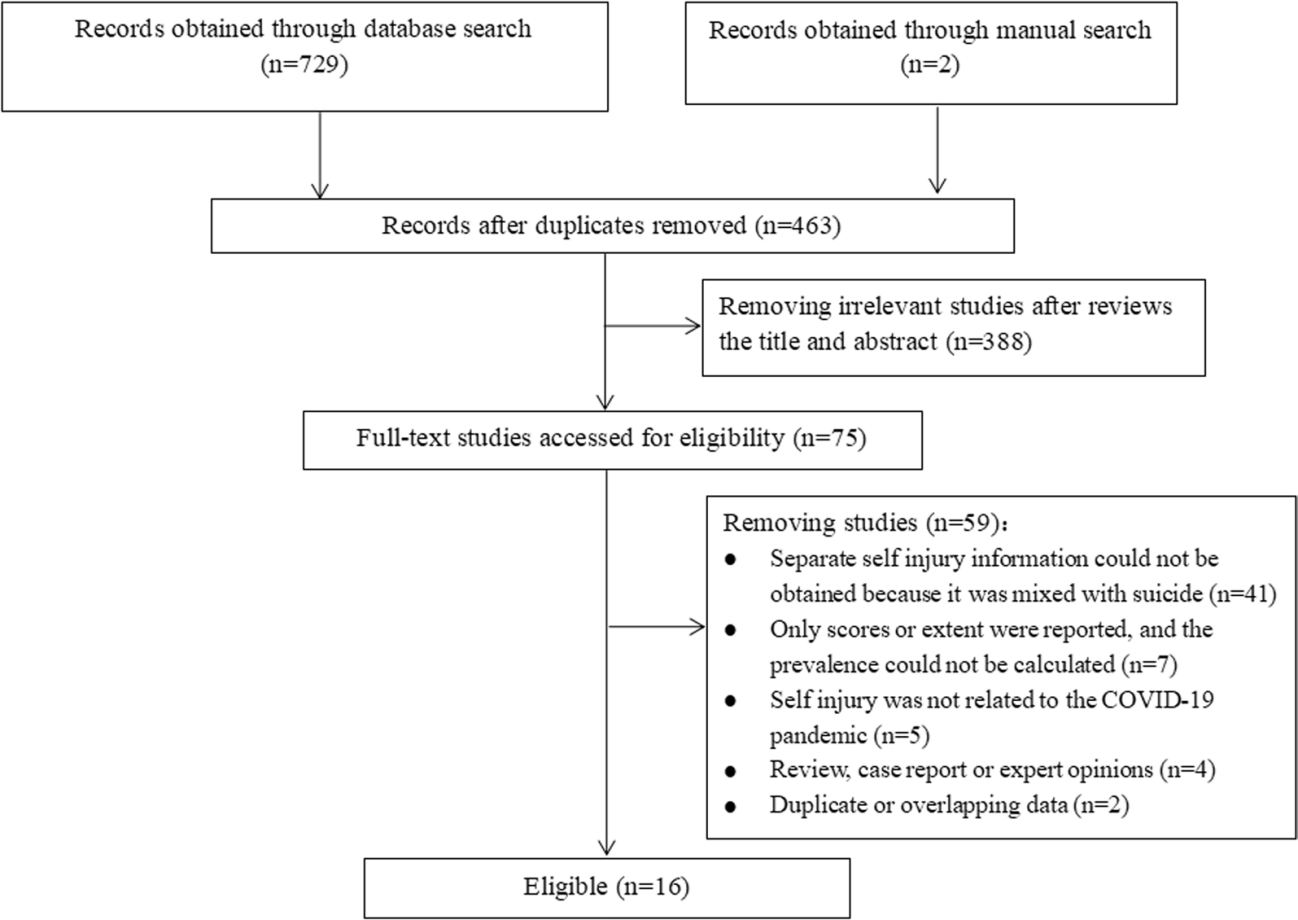
Flow diagram for study selection

After removing duplicate studies, 463 studies were retained. Next, we screened 463 studies at the title and abstract phase and excluded 388 irrelevant studies. Subsequently, the full texts of 75 studies that met the requirements were reviewed, of which 41 studies could not obtain separate self-harm information because it was mixed with suicide, 7 studies only reported the scores or extent of self-harm (e.g., mild, moderate and severe), and the prevalence could not be calculated due to the limited data on self-harm, 5 studies investigated self-harm that was not related to the COVID-19 pandemic, 4 studies were reviews, case reports or expert opinions, and 2 studies used duplicate or overlapping data. Finally, 16 eligible studies [23–38] were included in this meta-analysis, and the reasons for exclusion and details of study selection are given in Fig. 1.

### Characteristics of qualified studies

As displayed in Table 1, this meta-analysis included 16 studies published between 2020 and 2022, with sample sizes ranging from 228 to 49,227 (including 71 to 21,929 males and 127 to 22,846 females). Sixteen eligible studies were published from Asia (China, Korea, India), Europe (England, Italy, Switzerland), America (Mexico, US, Canada) and Oceania (New Zealand, Australia). Most of the included studies were carried out in the first half of 2020, with a few studies extending into the first half of 2021. In terms of study design and sample source, the majority of the included studies were cross-sectional or longitudinal studies (14/16), and more than half of the samples came from hospitals and schools (9/16). The included studies employed different, explicit and validated assessment tools, including one item to assess self-harm, electronic medical record, psychopathological interview or assessment, questionnaire or scale. Most importantly, all 16 eligible studies reported the number or occurrence of self-harm, of which 5 studies reported NSSI. Eleven of the studies included contained stratified variables such as gender, mental symptoms and restrictions.

**Table 1:** Characteristics of included studies describing self-harm

Author/year	Country	Study time	Study design	Sample source	Assessment tool	Age (years)	Sample size (male/female)	Number (N)		Stratifcation variables	Quality score
								SH	NSSI		
Iob E/2020	England	Mar 21–Apr 10, 2020	Cross-sectional	General population	One item that assessed self harm	≥ 18	44,775 (21,929/22846)	2174	NR	Gender; Mental symptoms; Restrictions	8
Du N/2021	China	Prior to Jan 30, 2021	Cross-sectional	Psychiatric hospital	Psychiatric interview that assessed NSSI	≤ 18	609 (77/532)	NR	266	Mental symptoms	6
Hawton K/2021	England	Mar 23–May 05, 2020	Cross-sectional	General hospital	Psychosocial assessments that evaluated self-harm	≥ 18	228 (101/127)	107	NR	Gender	5
Hermosillo AE/2021	Mexico	Nov 09–Dec 10, 2020	Cross-sectional	Schools	Suicidal Behaviors Schedule	14 ~ 21	8033 (3910/4123)	454	NR	Gender; Mental symptoms	4
Joyce LR/2021	New Zealand	Mar 26–Apr 28, 2020	Cohort	General hospital	Electronic medical record	0 ~ 60	935(396/539)	71	NR	Mental symptoms; Restrictions	5 *
Kim IH/2021	Korea	Aug 14–Sep 22, 2020	Cross-sectional	Schools	Self-Harm Inventory	22.91 ± 2.16	234 (71/163)	NR	78	Gender	5
Menculini G/2021	Italy	Jun 01, 2020–Jan 31, 2021	Cross-sectional	General hospital	Psychiatric visit that collected NSSI data	42.44 ± 16.42	447 (179/268)	NR	31	Mental symptoms	3
Paul E/2021	England	Apr 1, 2020–May 17, 2021	Longitudinal	General population	One item that assessed self harm	≥ 18	49,227 (NR)	3741	NR	NR	7
Robillard CL/2021	Canada	Jun 17–Jul 31, 2020	Cross-sectional	General population	Ontario Child Healthy Study Scales	12–18	809 (356/453)	256	NR	NR	6
Steinhoff A/2021	Switzerland	Apr–Sep, 2020	Longitudinal	Schools	One item that assessed self injury	≤ 22	786 (328/458)	57	NR	NR	7
Sugg MM/2021	US	Mar 13–Jul 20, 2020	Cross-sectional	Frontline workers and their children	Daily text conversations	13–65	32,580 (NR)	5150	NR	NR	4
Sveticic J/2021	Australia	Mar–Aug, 2020	Cross-sectional	Emergency departments of hospital	Electronic medical record	< 18	3190 (1504/1683)	482	NR	NR	4
Warne N/2021	England	May 26–Jul 04, 2020	Cohort	General population	Items adapted from the Child and Adolescent Self-Harm in Europe study	27–29	2657 (766/1891)	185	131	Restrictions	6 *
Wei Z/2021	China	Apr 10–20, 2020	Cross-sectional	Schools	NSSI questionnaire	15 ~ 23	1955 (1005/950)	NR	486	Gender	4
Bhattaram S/2022	India	Mar 24–Jun 30, 2020	Cross-sectional	Emergency departments of hospital	Electronic medical record	32.39 ± 12.06	828 (577/251)	102	NR	Gender; Mental symptoms	5
Slemon A/2022	Canada	May14–19, 2020 Sep 14–21, 2020	Longitudinal	General population	One item asking the history of self injuring behavior in the past two weeks	≥ 18	5993 (NR)	97	NR	Restrictions	4

*SH* self-harm; *NSSI* Non-suicidal self injury; *NR* Not reported

^*^Quality assessment by the Newcastle–Ottawa Scale (NOS)

### Assessment of methodological quality

The quality of 16 eligible studies was assessed by 11-item checklists for assessing observational studies recommended by the Agency for Healthcare Research and Quality (AHRQ) and the Newcastle‒Ottawa Scale (NOS) for assessing cohort studies, and the results are shown in Table 1. In observational studies, the quality score of 14 studies reached 3–8 points, with most of them having moderate methodological quality (12/14). Actually, the common problems in eligible observational studies were the lack of description of the inclusion criteria, the lack of treatment of the subjective factors of the evaluators affecting the study, the lack of reports on the causes and potential effects of data loss, and the lack of measures to assess or control confounders. In cohort studies, the quality score of 2 studies reached 5 and 6 points, respectively, above the low level of methodological quality. Two studies already had primary outcomes at the beginning and did not control for confounders, so these items were not scored.

### Merge of effect size

There were 16 datasets from 16 studies that provided suitable data for overall meta-analysis. In particular, the study sample of Sugg et al. [33] consists of two groups of samples: essential workers and children of essential workers. In Slemon’s [38] study, participants in both rounds included sexual and gender minority (SGM) identity and non-SGM identity. Joyce et al. [27] included two cohorts reporting the prevalence of self-harm before and during the COVID-19 lockdown. Under this circumstance, we treated those as merged datasets, and targeted and separate subgroup analyses were carried out as much as possible. Notably, Warne's cohort study [35] investigated the prevalence of self-harm before and during COVID-19, and data on self-harm during COVID-19 were employed. Warne's study [35] reported not only the total amount of self-harm without a specific purpose but also NSSI. The above two pieces of information were used for subgroup analysis based on the purpose of self-harm. The total sample size in these studies was 153,286.

**Fig. 2:**
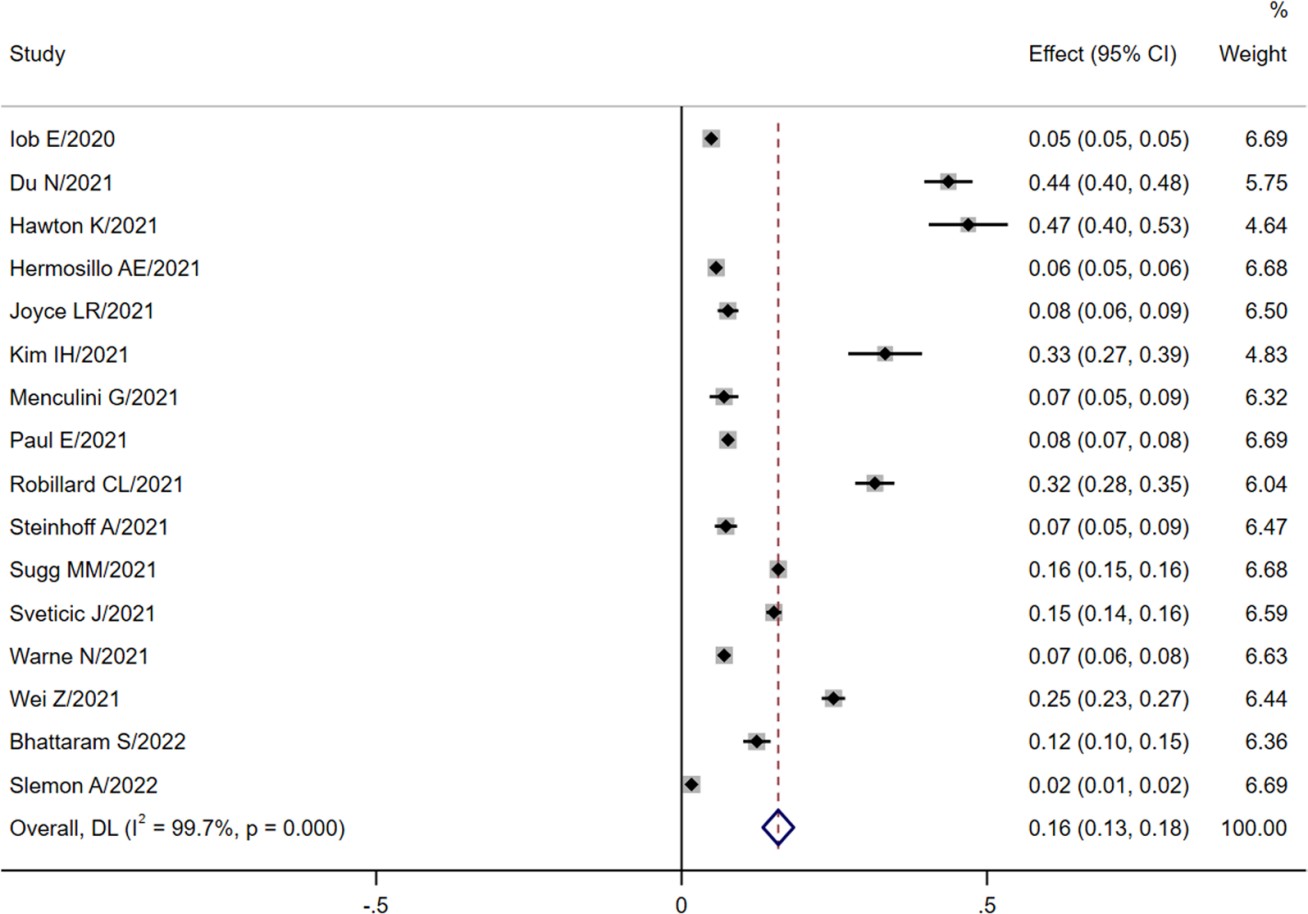
The global pooled prevalence of self-harm without logit transformation

As expected, the pooled estimates of the prevalence of self-harm showed a high level of heterogeneity (*I*^2^ > 99%, *P* < 0.005). Therefore, the random effect models was used to pool effect sizes. The pooled prevalence of self-harm in these studies was 15.8% (95%CI 13.3 ~ 18.3), as detailed in Fig. 2. The logit transformation resulted in a prevalence of 14.6% (CI 11.2 ~ 18.5). Based on the subgroup analysis and the transformed double arcsine method, the pooled prevalence of self-harm for different study places was 27.7% (95% CI 15.9 ~ 41.3) in Asia, 10.3% (95% CI 7.8 ~ 13.0) in Europe, 11.3% (95% CI 3.6 ~ 22.6) in America, and 11.2% (95% CI 4.9 ~ 19.5) in Oceania. Furthermore, the pooled prevalence of self-harm was 14.9% (95% CI 8.8 ~ 21.4) prior to July 2020 and 14.5% (95% CI 10.2 ~ 20.3) after July 2020. Meanwhile, cross-sectional studies had a higher prevalence of 18.4% (95% CI 14.1 ~ 23.1). The prevalence from different sample sources was as follows: school 16.0% (95% CI 5.5 ~ 30.6), hospital 15.6% (95% CI 7.5 ~ 26.0), and other source 13.2% (95% CI 8.6 ~ 18.6). The pooled prevalence of self-harm was 22.9% (95% CI 14.7 ~ 31.0) in adolescents and 11.7% (95% CI 8.0 ~ 15.5) in other age groups. The self-harm prevalence was 16.0% (95% CI 9.4 ~ 24.0) in male respondents and 20.6% (95% CI 13.0 ~ 29.3) in female respondents. Additionally, the pooled prevalence of NSSI was 20.5% (95% CI 7.4 ~ 38.0), compared with 11.6% (95% CI 8.3 ~ 15.3) for the unspecified purpose of self-harm. The pooled prevalence of self-harm among respondents with and without mental symptoms was 14.6% (95% CI 7.3 ~ 23.8) and 3.6% (95% CI 1.8 ~ 5.9), respectively. Notably, respondents who were restricted had a prevalence of 14.9% (95% CI 10.2 ~ 21.4), while respondents who were not restricted had a prevalence of 14.9% (95% CI 10.2 ~ 21.4), all of which are shown in Table 2.

**Table 2:** Subgroup analysis for the prevalence of self-harm

Variables	Prevalence, % (95% CI)	*P* value*
Study place		
Asia	0.277 (0.159–0.413)	*P* < 0.001
Europe	0.103 (0.078–0.130)	*P* < 0.001
America	0.113 (0.036–0.226)	*P* < 0.001
Oceania	0.112 (0.049–0.195)	*P* < 0.001
Study time		
Prior to July 2020	0.149 (0.088–0.214)	*P* < 0.001
After July 2020	0.145 (0.102–0.203)	*P* < 0.001
Study design		
Cross-sectional	0.184 (0.141–0.231)	*P* < 0.001
Non cross-sectional	0.055 (0.019–0.107)	*P* < 0.001
Sample source		
School	0.160 (0.055–0.306)	*P* < 0.001
Hospital	0.156 (0.075–0.260)	*P* < 0.001
Other sources	0.132 (0.086–0.186)	*P* < 0.001
Age		
Only adolescent	0.229 (0.147–0.310)	*P* < 0.001
All age groups	0.117 (0.080–0.155)	*P* < 0.001
Gender		
Male	0.160 (0.094–0.240)	*P* < 0.001
Female	0.206 (0.130–0.293)	*P* < 0.001
Purpose of self-harm		
Non-suicidal self-injury	0.205 (0.074–0.380)	*P* < 0.001
Unspecified	0.116 (0.083–0.153)	*P* < 0.001
Mental symptoms		
Yes	0.146 (0.073–0.238)	*P* < 0.001
No	0.036 (0.018–0.059)	*P* = 0.001
Restrictions		
Yes	0.112 (0.010–0.304)	*P* = 0.006
No	0.062 (0.032–0.100)	*P* < 0.001

^*^P value for*χ*^2^ statistic for heterogeneity

Overall, we estimated that studies with the following characteristics were more likely to report self-harm, including those conducted before July 2020, studies conducted in Asia, school/hospital studies, cross-sectional design, those related to self-harm, those involving female participants, those involving adolescent participants, and those involving participants with mental symptoms or restrictions.

### Sensitivity analysis

**Fig. 3:**
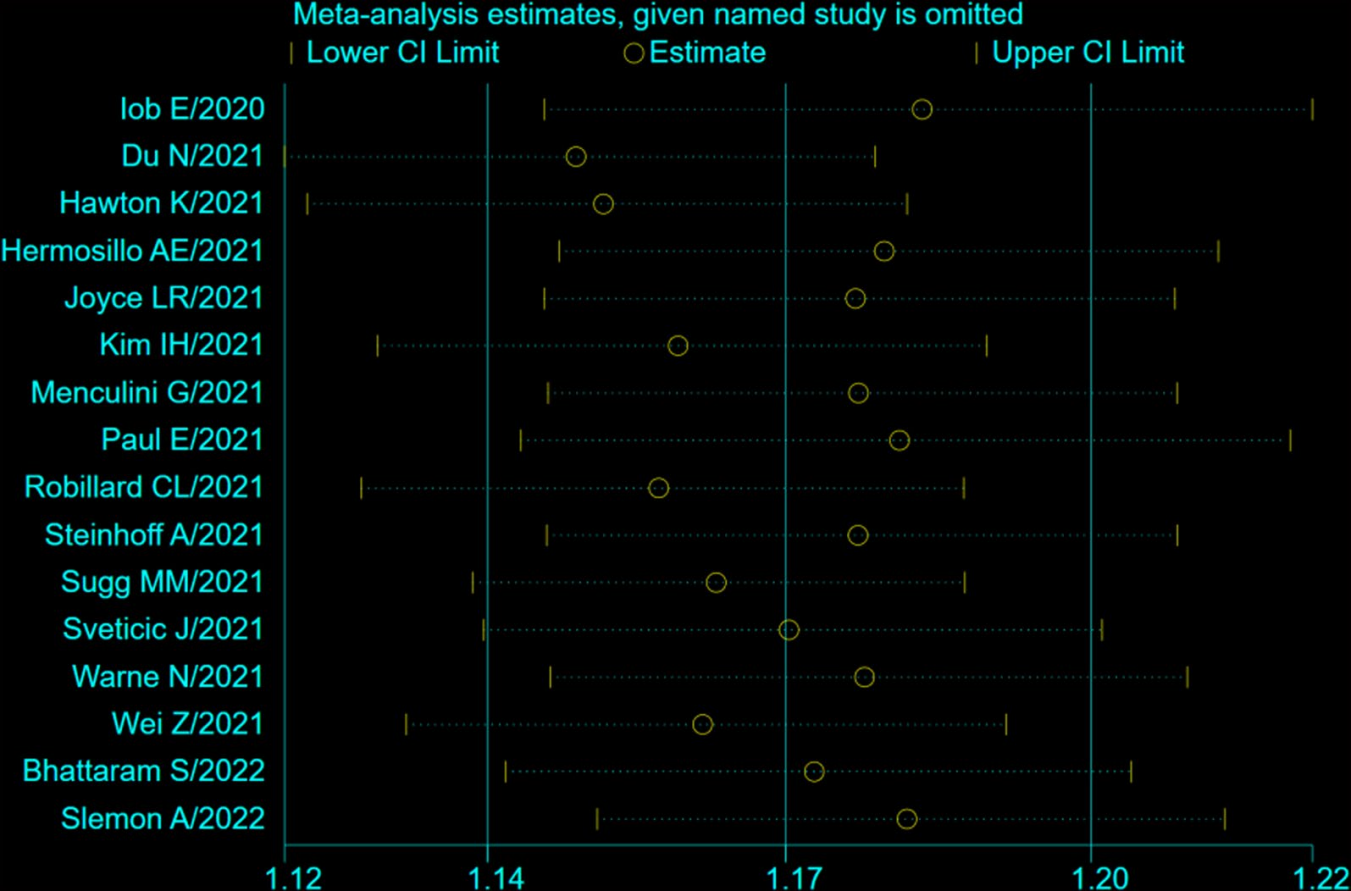
The results of sensitivity analysis

To explore the robustness and reliability of the results, we conducted sensitivity analysis based on the combined results of the meta-analysis. By gradual exclusion of each study, a sensitivity analysis was performed. Encouragingly, there was no significant change in the overall prevalence of self-harm after excluding the included studies one by one, indicating that the results of this meta-analysis were relatively stable and robust, as shown in Fig. 3.

## Discussion

The purpose of this review was to estimate the global prevalence of self-harm during COVID-19. We combined the data from 16 studies on self-harm-related COVID-19. The obtained pooled prevalence of self-harm suggested that self-harm closely related to COVID-19 cannot be ignored, and the characteristics included in the studies would affect the pooled prevalence of self-harm.

### The pooled prevalence of self-harm

The pooled prevalence of self-harm for all participants in our meta-analysis was 14.6%, higher than the 8.2% rate of self-harm obtained by Moller et al. [39] when they surveyed 4126 participants in 2013. Although the characteristics of the study site and population will play a role in the difference in the incidence of self-harm between the two studies, the effect of neoconiosis cannot be ignored. As is known to all, the outbreak of COVID-19 has had a great impact on people's work and life in a short time [4], which has acted as the source of recent stress greatly contributing to individual stress [40] and can be coordinated with self-harm persistent risk factors (impulsivity, adverse childhood experiences, etc.) to strengthen the demand for self-harm to alleviate stress and achieve rapid emotional release [41, 42]. Moreover, the pooled prevalence of self-harm in this review was slightly lower than that reported in the meta-analysis by Gillies et al. [43], which may be related to the fact that Gillies’s study was conducted among adolescents. Notably, self-harm is more common in adolescents than in other age groups [44]. Moreover, our pooled prevalence of self-harm was much lower than the prevalence of lifelong self-harm investigated by Muller et al. [45]. The studies included in this review were all related to COVID-19 outbreaks within a short period of time. However, the prevalence of lifelong self-harm means that other events in an individual's life rather than just a certain emergency may stimulate his desire to harm himself. In addition, the impact of a certain event on individuals may be revealed after a long period of time, which suggests that we should be concerned about the long-term impact of COVID-19 on self-harm [46].

### Subgroup analysis of variables

As expected, the pooled prevalence of self-harm in Asia was higher than that in other continents, which reflected the impact of COVID-19's sudden and explosive nature on self-harm. Our findings were consistent with previous studies that found that adverse mental health effects (i.e., self-harm) has been observed worldwide, notably in the Asia Pacific region, dominantly in countries such as China, where the first COVID-19 case was reported in 2019 [47–49]. As the first continent to discover COVID-19 and continue to spread, Asia is the first to feel the uncertainty and threatening nature of the epidemic [49]. People in Asian countries affected by the epidemic will undoubtedly shoulder the psychological burden caused by the epidemic [40], while people from other continents will buffer the stress for a certain period of time. This may explain the higher prevalence of self-harm in Asia during COVID-19. With regard to study time, we observed that studies conducted prior to July 2020 had a slightly higher prevalence of self-harm than studies conducted after July 2020. The psychological impact on individuals in the early stage of COVID-19 may be more significant than that in the later stage due to its uncertainty, severity, and persistence [50], increasing the possibility that individuals resort to self-harm to relieve negative pressure [25]. In addition, Patwary et al. [51] found that some social media in early COVID-19 may disclose unconfirmed COVID-19 information, which will not only eliminate public doubts about the epidemic but also aggravate psychological burden, especially for adolescents with a low ability to distinguish the authenticity of social media [52], who are the high-risk group of self-harm [44]. Unfortunately, due to the limitations of the included studies, we used July 2020 as a time dividing point to describe the different stages of development of COVID-19, but this time may not be representative, so the prevalence of the two time periods did not show a particularly significant difference.

In terms of study design, the pooled prevalence of self-harm was higher when the included cross-sectional studies were combined. The descriptive data obtained from cross-sectional studies are collected at a certain time point or in a short time interval [53], which objectively reflects the data characteristics of this time point. In this study, a cross-sectional design was used to collect data only during COVID-19, which indicated the change in the prevalence of self-harm due to COVID-19. It should be noted that the long-term impact of COVID-19 on self-harm also requires other types of study designs (i.e., cohort studies). Furthermore, samples from hospitals or schools may be more likely to harm themselves, which was confirmed by another study [54]. The reason for this may be discussed through the following explanations. Samples from hospitals may be affected by the disease, which may lead to anxiety in the recovery of the disease and the acquisition of regular treatment during COVID-19, especially patients with mental disorders who are easily influenced by the outside world and are unlikely to respond positively [55]. The trend of the prevalence of self-harm with age can be reflected by the samples from schools; that is, adolescents may be more prone to self-harm.

Notably, studies that included only adolescents reported a higher prevalence of self-harm than studies that covered all age groups, which was supported by other studies [43, 56]. Adolescence is a vulnerable phase for developing self-harm, as elevated levels of impulsivity and emotional reactivity are present due to brain developmental processes [57], so adolescents have a weak ability to control their own emotions and are prone to adopt self-harm due to external influences such as COVID-19. Consistent with previous studies [57, 58], our results indicated that the prevalence of self-harm among females was higher than that among males. Females are more likely to engage in self-harm due to inner emotional factors (e.g., “I felt very depressed”, “to escape painful memories”), while males are more likely to engage in self-harm for interpersonal reasons (e.g., “it makes me more gregarious”, “to makes me more masculine”) [59, 60]. It is clear that COVID-19 has brought more negative inner experiences and negative emotions to individuals. Additionally, self-harm among males focuses on social rather than emotional factors, and they have other strategies to achieve their goals rather than self-harm, including aggression and alcoholism [58].

In particular, we performed subgroup analysis according to the purpose of self-harm. NSSI may be more prone to occur. The self-harm literature is increasingly moving toward a separation of suicidal and NSSI, as outlined in the Diagnostic and Statistical Manual of Mental Disorders-Fifth Edition (DSM-5) [61]. Possible explanations for the high prevalence of NSSI should start from the special functions of NSSI. On the one hand, NSSI has a low-cost and immediate effect in eliminating unpleasant emotional states [62], while COVID-19's outbreak and popularity are often accompanied by negative emotions, including fear, sadness, tension and anxiety and despair [63]. On the other hand, influenced by COVID-19's infectivity, remote life, virtual classrooms and lockdown are the main lifestyles to maintain physical and social distance [64], which seriously affects social interaction and increases feelings of emptiness and loneliness. NSSI can provide stimulation by experiencing strong emotions and eliminating feelings of emptiness and loneliness [63].

In accordance with other studies [54, 62], we found that respondents with mental symptoms (depressive, anxiety symptoms, etc.) reported a higher prevalence of self-harm than those without mental symptoms. Likewise, previous studies have also shown that the main risk factors for self-harm include accompanying mental symptoms, especially mental illness involving mood disorders [54]. It is well known that psychiatric patients with emotional regulation disorders have difficulty in regulating negative emotions caused by negative events, such as the inability to cope with negative emotions caused by COVID-19 [55], while self-harm has been proven to be a coping strategy that can regulate emotions [54]. Hence, careful consideration by caregivers and healthcare system adaptations to allow for mental health support should be required to reduce the risk behaviors of patients with mental symptoms despite the restrictions of COVID-19.

Finally, we separately estimated the pooled prevalence of self-harm in both groups based on whether restrictions were applied in post-COVID-19 studies, suggesting that the group with restrictions may be more likely to report a higher prevalence of self-harm. To abate the rate of infection, global governments have imposed restrictions to some extent, including restrictions on social activities, shopping, and exercise [65]. The introduction of limits is likely to have a detrimental influence on mental health and well-being, as we all know that social connection is vital in giving psychological support and help [65]. It should be emphasized that objective social isolation and subjective loneliness are associated with a higher prevalence of self-harm [66]. Notably, many patients and their families forgo or delay health care due to fear or decreased access to medical services during the lockdown [67], which is not conducive to the rehabilitation of patients and increases their anxiety and worry, especially patients with chronic illnesses such as mental disorders. In brief, the employment of restrictions during COVID-19 may exacerbate negative emotions and worsen them, which may be an incentive for individuals to engage in self-harm. This suggests that multifunctional social software, home exercise programs, strategies to enhance relapse prevention and the use of alternative approaches such as e-health technologies need to be implemented [68].

### Sensitivity analysis

Based on sensitivity analysis, the results of this review were robust and reliable. Nevertheless it must be acknowledged that the majority of the included studies are in fact of medium quality. Since the emergence of COVID-19, more studies have focused on reducing the infection rate and treating diagnosed patients in a short period of time with limited resources, and the number of studies involving mental health (self-harm) has been limited. The majority of research in linked domains were in their early phases, which might have had an impact on the quality of the studies. In addition, the sudden outbreak of COVID-19 makes researchers eager to find the mental health outcomes of COVID-19 as soon as possible to take targeted measures as much as possible. Due to the inadequate consideration of study design or study scheme, it is likely to be detrimental to the study quality. Moreover, since the outbreak of COVID-19 has only lasted for approximately two years, all studies have failed to explore the long-term impact of the epidemic on self-harm, which will undoubtedly have an impact on the study quality. The impact of COVID-19 on individuals is profound and lasting, especially on mental health. As an important manifestation that is not conducive to mental health, self-harm is likely to be used by individuals to quickly regulate emotions and alleviate negative emotions. During COVID-19, further study on self-harm will be conducted, particularly in particular nations or ethnic groups. The current study may provide some insight into the current state of the field, which can help future studies in the field increase both in quantity and quality.

### Limitations

Many limitations should be acknowledged. First, because COVID-19 has only emerged for approximately two years and there were only a few relevant studies, most of the studies included were observational studies, and inherent biases and differences in the design of observational studies tend to increase the risk of heterogeneity. Second, although we incorporate data from a significant period during COVID-19, it would be useful to conduct a longitudinal study over longer time spans, as the antecedent factors for and outcomes of self-harm may change throughout the lifespan. Next, we performed subgroup analysis of relevant variables based on the literature and clinical experience, which may not include some variables that affect heterogeneity. The division of the study duration into multiple phases may result in less accurate results in the subgroup analysis of the study time. In addition, subgroup analysis did not completely solve or explain the obvious heterogeneity. Therefore, our findings should be cited with caution. Moreover, the quality assessment of the included studies was mostly at the medium level, which may affect the study results. Finally, this review was limited by language and region and did not include non-English or non-Chinese studies. Maybe there is some available information.

### Implications

Despite the above limitations, our findings have implications for policy and practice. In contrast to previous studies that concentrate on the effect of COVID-19 on mental health, this research, to a certain degree, focuses on a particular subject (self-harm), attracting the attention of governments all over the globe and encouraging the wise use of resources. Most strikingly, the pooled prevalence of self-harm during COVID-19 in this review was not cheerful. Therefore, it also suggests that relevant departments should formulate relevant preventive measures in time to identify high-risk factors for self-harm as soon as possible, such as adolescents, females, groups with mental symptoms or groups with loneliness and emptiness after experiencing restrictive measures. By creating targeted public health intervention measures, such as regular psychological assessment of the aforementioned high-risk groups through the combination of online and offline interventions to strengthen social interaction, relevant departments can lessen the negative effects of these risk factors on individual self-mutilation. For instance, studies have confirmed that brief contact interventions (i.e., telephone/letter/postcard contact and emergency green cards) may contribute to enhancing social support and social contact in a long-distance context to reduce the prevalence of self-harm [69]. More importantly, timely psychological counseling should not be neglected in response to self-harm that has already occurred, and professional medical treatment can be considered when necessary.

Four directions for further research are emphasized. First, future studies can determine the comparable and long-term impact of COVID-19 on the prevalence of self-harm by establishing an appropriate control group and adequate follow-up. Second, the studies included in this study only represented the situation of self-harm in a few countries, which was considered to be jointly shaped by cultural and social environment. Therefore, there may be differences in the impact of COVID-19 on different countries, which requires reasonably designed studies in different countries in the future, especially in low- and middle-income countries. Third, we should not stop exploring the variables affecting heterogeneity for further subgroup analysis. Some variables that may be suitable for subgroup analysis have been reported in only one study or have not been reported in any included study, and subgroup analysis cannot be carried out. For example, the study of Iob et al. [23] stratified the number of self-harm in the sample according to the COVID-19 diagnosis, which was the only study to report the COVID-19 diagnosis. Recent evidence has indicated that individuals with a diagnosis of COVID-19 have serious traumatic experiences and adverse mental health [12, 23]. If conditions allow, it is necessary to conduct psychological evaluation on individuals diagnosed with COVID-19 to find more specific and special connections. Finally, large-sample, high-quality studies need to be conducted, which are not only limited to the estimation of the prevalence of self-harm but also focus on the risk factors and prevention/intervention strategies of self-harm to broaden the research field of relevant studies to suggest ways by which the levels of self-harm can be reduced.

## Conclusions

The current study is an initial step in exploring the impact of COVID-19 on self-harm worldwide. The prevalence of self-harm was estimated by utilizing a merger of studies on self-harm during COVID-19 worldwide. In doing so, we initially learned about self-harm that is closely related to COVID-19 around the world, although there is significant heterogeneity among the studies. In general, the results of the meta-analysis showed that the pooled prevalence of self-harm during COVID-19 was not optimistic, and the prevalence of self-harm was different by subgroup analysis based on the variables, including study place, study time, age, gender, study design, purpose of self-harm, mental symptoms and restrictions. These conclusions, albeit sobering, help to explore targeted prevention and intervention strategies and justify new and exciting future directions in related studies. For example, these findings are beneficial to arouse social attention to mental health (i.e., self-harm), especially during COVID-19. The identification of high-risk groups, the opening of psychological counseling channels, and the implementation of social contact or other interventions are crucial. In the meantime, it is of great significance for future studies to promote the refinement and characterization of samples to find more valuable evidence in which the variables involved in subgroup analysis can be taken into account.

## Supplementary Information

**Additional file 1.** Full search strategy.

## Data Availability

The datasets generated and/or analysed during the current study are not publicly available due to the existence of unpublished papers but are available from the corresponding author on reasonable request.
