# Supplementary material for: Global prevalence of self-harm during the COVID-19 pandemic: a systematic review and meta-analysis
Source: BMC Psychol. 2023 May 5;11:149. doi: 10.1186/s40359-023-01181-8 (PMC10160734; doi:10.1186/s40359-023-01181-8)
Supplement: Supplementary file 1 — Additional file 1. Full search strategy. [file 40359_2023_1181_MOESM1_ESM.docx]

**Supplementary material-Full search strategy**

|  | | | | | |
| --- | --- | --- | --- | --- | --- |
| **#** | **Database** | **Search term** | **Mid-June 2021 Results** | **Jan 2022 rerun** | **Results added to totals** |
| 1 | Medline | (Coronavirus OR "2019-nCov" OR "2019nCov" OR "covid-19" OR "Sars-Cov-2").ti,ab | 52314 | 63211 |  |
| 6 | Medline | (Coronavirus OR 2019-nCov OR 2019nCov OR covid-19 OR Sars-Cov-2 OR pandemic OR "novel coronavirus 19").ti,ab | 62710 | 86744 |  |
| 7 | Medline | exp CORONAVIRUS/ | 18264 | 35682 |  |
| 3 | Medline | "SELF-INJURIOUS BEHAVIOR"/ OR "SELF MUTILATION"/ | 13621 | 18956 |  |
| 10 | Medline | (self-harm OR "self harm" OR NSSI OR "non-suicidal self injury" OR self-injur* OR "self injur*" OR "self mutilat*" OR "self inflicted" OR "self destructive" OR "nonsuicidal self injury").ti,ab | 112136 | 109737 |  |
| 16 | Medline | (1 OR 6 OR 7)  [ALL coronavirus terms] | 71866 | 92653 |  |
| 17 | Medline | (3 OR 10)  [ALL self-harm terms] | 138960 | 124278 |  |
| 18 | Medline | (16 AND 17)  [ALL coronavirus AND ALL self-harm] | 168 | 274 |  |
| 19 | Medline | 18 [DT 2019-2022] | 78 | 165 | 122 saved |
| 22 | EMBASE | (Coronavirus OR 2019-nCov OR 2019nCov OR covid-19 OR Sars-Cov-2 OR pandemic OR "novel coronavirus 19").ti,ab | 62342 | 90226 |  |
| 23 | EMBASE | exp CORONAVIRINAE/ | 16316 | 20425 |  |
| 24 | EMBASE | (22 OR 23)  [ALL coronavirus terms] | 75812 | 100164 |  |
| 25 | EMBASE | AUTOMUTILATION/ | 19321 | 19527 |  |
| 26 | EMBASE | (self-harm OR "self harm" OR NSSI OR "non-suicidal self injury" OR self-injur* OR "self injur*" OR "self mutilat*" OR "self inflicted" OR "self destructive" OR "nonsuicidal self injury").ti,ab | 100142 | 101157 |  |
| 27 | EMBASE | (25 OR 26)  [ALL self-harm terms] | 123346 | 131209 |  |
| 28 | EMBASE | (24 AND 27)  [ALL coronavirus AND ALL self-harm] | 142 | 296 |  |
| 29 | EMBASE | 29 [DT 2019-2022] | 72 | 181 | 115 saved |
| 30 | PsycINFO | (Coronavirus OR 2019-nCov OR 2019nCov OR covid-19 OR Sars-Cov-2 OR pandemic OR "novel coronavirus 19").ti,ab | 2472 | 3216 |  |
| 31 | PsycINFO | PANDEMICS/ | 710 | 1215 |  |
| 32 | PsycINFO | (30 OR 31)  [ALL coronavirus terms] | 3214 | 3752 |  |
| 33 | PsycINFO | "SELF-INJURIOUS BEHAVIOR"/OR "SELF-MUTILATION"/ | 30152 | 34070 |  |
| 34 | PsycINFO | (self-harm OR "self harm" OR NSSI OR "non-suicidal self injury" OR self-injur* OR "self injur*" OR "self mutilat*" OR "self inflicted" OR "self destructive" OR "nonsuicidal self injury).ti,ab | 51288 | 53641 |  |
| 35 | PsycINFO | (33 OR 34)  [ALL self-harm terms] | 60121 | 62336 |  |
| 36 | PsycINFO | (32 AND 35)  [ALL coronavirus AND ALL self-harm] | 85 | 142 |  |
| 37 | PsycINFO | 36 [DT 2019-2022] | 36 | 68 | 52 saved |
| 38 | Web of Science | (Coronavirus OR 2019-nCov OR 2019nCov OR covid-19 OR Sars-Cov-2 OR pandemic OR "novel coronavirus 19").ti,ab | 64717 | 68236 |  |
| 39 | Web of Science | exp CORONAVIRINAE/ | 15328 | 19113 |  |
| 40 | Web of Science | (38 OR 39)  [ALL coronavirus terms] | 66383 | 79410 |  |
| 41 | Web of Science | "SELF-INJURIOUS BEHAVIOR"/ OR "SELF MUTILATION"/ | 12051 | 15422 |  |
| 42 | Web of Science | (self-harm OR "self harm" OR NSSI OR "non-suicidal self injury" OR self-injur* OR "self injur*" OR "self mutilat*" OR "self inflicted" OR "self destructive" OR "nonsuicidal self injury").ti,ab | 84316 | 86745 |  |
| 43 | Web of Science | (41 OR 42)  [ALL self-harm terms] | 96278 | 92357 |  |
| 44 | Web of Science | (40 AND 43)  [ALL coronavirus AND ALL self-harm] | 114 | 158 |  |
| 45 | Web of Science | 44 [DT 2019-2022] | 89 | 102 | 85 saved |
| 46 | PubMed | (Coronavirus OR 2019-nCov OR 2019nCov OR covid-19 OR Sars-Cov-2 OR pandemic OR "novel coronavirus 19").ti,ab | 70129 | 81433 |  |
| 47 | PubMed | exp CORONAVIRINAE/ | 17246 | 18434 |  |
| 48 | PubMed | (46 OR 47)  [ALL coronavirus terms] | 75639 | 86595 |  |
| 49 | PubMed | "SELF-INJURIOUS BEHAVIOR"/OR "SELF-MUTILATION"/ | 16726 | 17345 |  |
| 50 | PubMed | (self-harm OR "self harm" OR NSSI OR "non-suicidal self injury" OR self-injur* OR "self injur*" OR "self mutilat*" OR "self inflicted" OR "self destructive" OR "nonsuicidal self injury").ti,ab | 135582 | 141217 |  |
| 51 | PubMed | (49 OR 50)  [ALL self-harm terms] | 141010 | 158368 |  |
| 52 | PubMed | (48 AND 51)  [ALL coronavirus AND ALL self-harm] | 166 | 189 |  |
| 53 | PubMed | 52 [DT 2019-2022] | 115 | 123 | 146 saved |
| 54 | Cochrane | (Coronavirus OR 2019-nCov OR 2019nCov OR covid-19 OR Sars-Cov-2 OR pandemic OR "novel coronavirus 19").ti,ab | 1215 | 2466 |  |
| 55 | Cochrane | exp CORONAVIRINAE/ | 251 | 389 |  |
| 56 | Cochrane | (54 OR 55)  [ALL coronavirus terms] | 1316 | 2530 |  |
| 57 | Cochrane | "SELF-INJURIOUS BEHAVIOR"/OR "SELF-MUTILATION"/ | 2421 | 3002 |  |
| 58 | Cochrane | (self-harm OR "self harm" OR NSSI OR "non-suicidal self injury" OR self-injur* OR "self injur*" OR "self mutilat*" OR "self inflicted" OR "self destructive" OR "nonsuicidal self injury").ti,ab | 4936 | 5408 |  |
| 59 | Cochrane | (57 OR 58)  [ALL self-harm terms] | 5108 | 6124 |  |
| 60 | Cochrane | (56 AND 59)  [ALL coronavirus AND ALL self-harm] | 34 | 48 |  |
| 61 | Cochrane | 60 [DT 2019-2022] | 8 | 11 | 18 saved |
| 62 | CNKI | (新冠 OR 新冠病毒 OR 新冠肺炎 OR 新型冠状病毒 OR 新冠疫情).ti,ab | 48450 | 52136 |  |
| 63 | CNKI | (自伤 OR 自残 OR 非自杀性自伤 OR 自我伤害 OR 故意自残).ti,ab | 26515 | 37423 |  |
| 64 | CNKI | (62 AND 63)  [ALL coronavirus AND ALL self-harm] | 235 | 368 |  |
| 65 | CNKI | 64 [DT 2019-2022] | 95 | 138 | 112 saved |
| 66 | Wangfang | (新冠 OR 新冠病毒 OR 新冠肺炎 OR 新型冠状病毒 OR 新冠疫情).ti,ab | 28432 | 40218 |  |
| 67 | Wangfang | (自伤 OR 自残 OR 非自杀性自伤 OR 自我伤害 OR 故意自残).ti,ab | 16150 | 21424 |  |
| 68 | Wangfang | (66 AND 67)  [ALL coronavirus AND ALL self-harm] | 187 | 220 |  |
| 69 | Wangfang | 64 [DT 2019-2022] | 82 | 138 | 79 saved |
